# Unsupervised Learning‐based Drug Repositioning for Alzheimer’s Disease Targeting Brain Cell Type Specific Genetic Subtypes

**DOI:** 10.1002/alz.090350

**Published:** 2025-01-09

**Authors:** Ankit Kumar Mishra, Nathan J Sahelijo, Dhawal Priyadarshi, Gyungah R Jun

**Affiliations:** ^1^ Boston University Chobanian & Avedisian School of Medicine, Boston, MA USA

## Abstract

**Background:**

Alzheimer’s disease (AD) presents challenges with its complex neurodegenerative mechanisms, leading to a high failure rate in clinical trials. While drug repositioning offers a cost‐effective solution, the lack of a subtype‐driven strategy hinders success. Previously, we defined genetic subtypes and their prioritized genes for each genetic subtype (Sahelijo et al., 2022). This study evaluated unsupervised learning algorithms to characterize existing compounds targeting prioritized genes for the genetic subtypes.

**Method:**

Compounds in at least Phase 2 clinical trials were gathered from PubChem. Active compounds against subtype‐specific genes were selected, and their structural data were transformed into pharmacophore fingerprints using ChemmineR. Unsupervised algorithms (Agglomerative Clustering, Ensemble Clustering, Gaussian Mixture Models, Bayesian Gaussian Mixture Models) were optimized using evaluation metrics (Calinski‐Harabasz, Davies‐Bouldin, Silhouette) to cluster compounds within each subtype with the optimal cluster number and optimal algorithm. The finalized clusters of compounds were evaluated using significance values and mean within‐cluster Jaccard similarity scores and were characterized with target genes and mechanisms of action.

**Result:**

Four of the nine genetic subtypes generated compound clusters, including 3 clusters using Agglomerative Clustering for Ast‐M2 with 11 targets and 180 compounds, 2 clusters using BGMM for Ast‐M9 with 14 targets and 341 compounds, and 4 clusters using Ensemble Clustering for Oli‐M45 with 11 targets and 66 compounds and for Oli‐M50 with 18 genes and 431 compounds. We observed common structural signatures between Ast‐M2, Ast‐M9, and Oli‐M50 clusters, while Oli‐M45 clusters did not share any signatures with other clusters. The most significant cluster was found for the Oli‐M45 subtype, with a cluster significance value of 8.49 and the highest mean compound similarity score of 0.96. The top‐ranked cluster primarily contained Vinblastine formulations—microtubule and tubulin polymerization inhibitors—targeting TUBA1A and TUBA1B.

**Conclusion:**

We demonstrated a novel drug repositioning framework for AD using unsupervised learning algorithms, enabling precision medicine and subtype‐driven repositioning. This framework will be implemented in our future software tools.

